# Activation of p53: How phosphorylated Ser15 triggers sequential phosphorylation of p53 at Thr18 by CK1δ

**DOI:** 10.1002/prot.26393

**Published:** 2022-07-14

**Authors:** Sonia T. Nicolaou, Srinivasaraghavan Kannan, Jim Warwicker, Chandra S. Verma

**Affiliations:** ^1^ Faculty of Biology, Medicine and Health, School of Biological Sciences Manchester Institute of Biotechnology, University of Manchester Manchester UK; ^2^ Bioinformatics Institute, Agency for Science, Technology, and Research (A*STAR) Singapore Singapore; ^3^ School of Biological Sciences Nanyang Technological University Singapore Singapore; ^4^ Department of Biological Sciences National University of Singapore Singapore Singapore

**Keywords:** fuzzy complex, intrinsically disordered proteins, molecular dynamics, p53, phosphorylation

## Abstract

The N‐terminal transactivation domain (TAD) of p53 is a disordered region with multiple phosphorylation sites. Phosphorylation at Thr18 is crucial for the release of p53 from its negative regulator, MDM2. In stressed cells, CK1δ is responsible for phosphorylating Thr18, but requires Ser15 to be phosphorylated. To understand the mechanistic underpinnings of this sequential phosphorylation, molecular modeling and molecular dynamics simulation studies of these phosphorylation events were carried out. Our models suggest that a positively charged region on CK1δ near the adenosine triphosphate (ATP) binding pocket, which is conserved across species, sequesters the negatively charged pSer15, thereby constraining the positioning of the rest of the peptide, such that the side chain of Thr18 is positioned close to the γ‐phosphate of ATP. Furthermore, our studies show that the phosphorylated p53 TAD1 (p53pSer15) peptide binds more strongly to CK1δ than does p53. p53 adopts a helical structure when bound to CK1δ, which is lost upon phosphorylation at Ser15, thus gaining higher flexibility and ability to morph into the binding site. We propose that upon phosphorylation at Ser15 the p53 TAD1 peptide binds to CK1δ through an electrostatically driven induced fit mechanism resulting in a flanking fuzzy complex.

## 1 INTRODUCTION

Proteins are essential components of the living cell whose functionality is highly controlled by post‐translational modifications (PTMs). Intrinsically disordered proteins and regions of proteins (simply referred to as IDPs) lack a fixed three‐dimensional (3D) structure and instead exist as dynamic ensembles of interchanging conformations.^1^, ^2^, ^3^, ^4^, ^5^ PTM sites are enriched in IDPs, and due to their flexible nature, they are easily accessible to modifying enzymes.^6^ Phosphorylation is one of the most common PTMs and phosphorylation sites are typically found in disordered regions.^7^ During a phosphorylation event, the neutral side chain –OH group of a Ser, Thr, or Tyr residue is modified to a negatively charged PO_4_^2−^ group by the transfer of a phosphate from an adenosine triphosphate (ATP) molecule bound to a protein kinase. The increase in negative charge from the addition of a phosphate group alters the steric, electrostatic, and hydrophobic properties of the protein, as well as its ability to interact with binding partners and thus is considered an important regulatory mechanism amongst eukaryotic processes,^6^, ^8^, ^9^ and to a lesser extent in prokaryotes.^10^

Tumor suppressor p53 is a transcription factor with ordered and disordered regions and serves as a hub in protein–protein interaction networks. It includes two large intrinsically disordered regions with multiple phosphorylation sites: an N‐terminal transactivation domain (TAD) and a C‐terminal regulatory domain.^11^ Nine out of the more than 20 phosphorylation sites in p53 are located in TAD.^12^ The TAD is divided into two subdomains: TAD1 (residues 1–40) and TAD2 (residues 41–61). In unstressed cells, TAD1 interacts with E3 ubiquitin ligase MDM2, keeping p53 levels low through continuous proteasomal degradation.^13^, ^14^, ^15^ Upon stress, a series of PTM events (mainly phosphorylation) are initiated, activating p53 by releasing it from MDM2. Free p53 is stabilized by CBP/p300 and is able to accumulate in cells and turn on the transcription of genes that suppress tumor activity.^16^, ^17^ Disordered TAD1 interacts with both MDM2 and CBP/p300 (TAZ1 and TAZ2 domains) and forms an amphipathic helix in the bound state.^12^, ^18^

Phosphorylation at Thr18 is crucial for the release of p53 from MDM2.^19^ This phosphorylation is carried out by casein kinase 1δ (CK1δ).^20^ The protein kinase CK1 family comprises a set of highly similar Ser/Thr kinases involved in regulating a variety of cellular functions including but not limited to the circadian rhythm, cellular response to DNA damage, and apoptosis.^21^ CK1 substrates often contain acidic or phosphorylated residues with a consensus sequence of pSer/pThr‐X‐X‐(X)‐Ser/Thr, where pSer/pThr are phosphorylated Ser or Thr (this position can also be occupied by an acidic residue), X represents any amino acid, and (X) represents a possible third residue.^22^, ^23^ Consistent with the sequence motif recognized by CK1, efficient phosphorylation of Thr18 in TAD1 requires prior phosphorylation at Ser15.^20^ Although p53 has been extensively studied over the years, mechanisms that underpin several functions of p53 still remain unknown, including why Ser15 phosphorylation is a prerequisite for Thr18 phosphorylation.

IDPs frequently fold upon binding to their binding partners, a phenomenon known as coupled folding and binding.^24^ This either occurs through conformational selection, where the bound‐state‐like structure of the IDP is present in the unbound state ensemble of conformations, or through induced fit, where the IDP folds into the desired state after the initial interaction with its binding partner.^25^, ^26^, ^27^ Often, IDPs bind to their partners through a combination of the two mechanisms. However, some IDPs remain disordered even after binding and form fuzzy complexes.^28^ Fuzzy complexes are divided into four structural categories: polymorphic, clamp, flanking, and random. Complexes are referred to as polymorphic when at least one of the partners adopts a few alternative structures (two in the simplest case).^28^, ^29^ Clamp complexes are formed when only the N‐ and C‐termini of the IDP interact with a partner, while the linker remains disordered due to a lack of permanent contacts.^28^ Flanking complexes on the other hand contain a short recognition motif within the linker region that is used for binding to the partner protein, while the termini of the IDP retain their conformational variability.^28^, ^29^ In random complexes, IDPs only interact with their partners through transient contacts and binding does not induce the formation of any secondary structure.^28^, ^29^ It is likely that the complex between the p53 TAD1 peptide and CK1δ falls into one of these categories.

To examine the effect of Ser15 phosphorylation on the dynamics of the p53 TAD1 peptide (henceforth referred to as p53 peptide) and its recognition by the kinase, we studied the conformational dynamics of a free p53 peptide as well as a p53 peptide in complex with CK1δ, in the presence and absence of Ser15 phosphorylation. In this study, the technique of accelerated molecular dynamics (aMD) was used to ensure comprehensive sampling of the conformational states of the systems.^30^ Our results suggest that the increase in negative charge due to Ser15 phosphorylation leads to high conformational flexibility in the p53 peptide in both its free and bound states. This enables the peptide to dock such that phosphorylated Ser15 is sequestered in a positively charged region of the kinase which then holds the remaining peptide, thus localizing Thr18 to the vicinity of the ATP with its hydroxyl oriented in position to accept the incoming phosphate from ATP. In the bound state, the p53pSer15 peptide loses its helical structure, whereas the p53 peptide remains partly helical even in the bound state. We speculate that the phosphorylated p53 peptide binds CK1δ through an induced fit mechanism to form a fuzzy complex.

## 2 MATERIALS AND METHODS

### 2.1 System preparation for free p53 peptides

An initial fully extended p53 TAD1 peptide, residues 9–24, without phosphorylation, was built using the xleap module of the AmberTools19^31^ package to avoid bias from bound structures. The N‐ and C‐termini of the peptide were capped with acetyl and *N*‐methyl groups, respectively.

### 2.2 System preparation for p53 peptides bound to CK1δ

The experimental structure of CK1δ was obtained from the Protein Data Bank (PDB id 6ru6; resolution 2.05 Å)^32^, ^33^ and all the crystallographic waters were retained. The binding pose of the peptide to CK1δ was taken from Protein Data Bank structure 6ru7, that includes a p63 peptide (resolution 2.08 Å).^32^ ATP and two complexed Mg^2+^ ions were added using Protein Data Bank entry 3x2w (resolution 1.7 Å)^34^ by superimposing the protein structures using PyMOL.^35^ Modeller^36^ was used to fill missing residues in the structure 6ru6. The C‐terminus of CK1δ was capped using an *N*‐methyl cap. The likely protonation states of hydrogens in the 6ru6 structure were generated using the programs MolProbity^37^ and PDB2QR.^38^ Two different p53 TAD1 peptides, residues 9–24, (a) without phosphorylation and (b) with Ser15 phosphorylation, bound to CK1δ with Thr18 residue placed adjacent to ATP, were created by mutating the peptide residues from PDB structure 6ru7 to p53 residues using PyMOL. The mutated peptides were added to the 6ru6 structure by superimposing them using PyMOL. The N‐ and C‐termini of the peptides were capped with acetyl and *N*‐methyl groups, respectively.

### 2.3 System preparation for mutant CK1δ complexes

The following residues of CK1δ in both the p53 and p53pSer15 complexes were mutated to Ala: Arg98, Lys130, Arg178, Lys221, Arg222, and Lys224. Mutations were determined from alanine scanning calculations. Since we were interested in the dominant interactions that the negatively charged peptide (due to phosphorylated Ser15) makes with the kinase, only the positively charged residues that contributed most to binding were mutated. PyMOL was used to perform the mutations to Ala.

### 2.4 Molecular dynamics simulations of free peptides

The linear free p53 peptide structure without phosphorylation was used as the starting structure for this study. Implicit solvent molecular dynamics (MD) simulations were carried out to collapse the extended structure created with xleap to reduce the size of the water box. The behavior of peptides in solution is simulated by immersing them in a box of water molecules and carrying out MD simulations. However, the extended structures of the peptides require a large water box and to reduce the associated computational costs, we initially subject the peptides to implicit solvent MD simulations to collapse their conformations, thereby requiring smaller boxes of water molecules for explicit solvent MD simulations.^39^ The sander module of the Amber11^40^ package was used for energy minimization and equilibration of the system with the Amber ff14SB^41^ force field. A 1500 step minimization was initially conducted, followed by gradual heating of the system from 0 to 300 K over 80 ps. This was followed by further heating of the system from 300 to 450 K over 5 ns in order to enhance conformational sampling. A production simulation at 300 K was carried out over 0.5 ns using pmemd, the CUDA module of Amber18.^42^

Next, the xleap module of AmberTools19 was used to prepare the system for explicit solvent MD simulations using the implicit solvent production model as the starting structure. The system was solvated in an octahedral box using the TIP3P water model,^43^ with at least 10 Å separating the solute atoms and the edges of the box. Counter ions were added to neutralize the system. Energy minimization and equilibration simulations were carried out using the sander module of the Amber11 package with the ff14SB force field. A 10 000 step energy minimization of the system was carried out, with positional restraints on the peptide (force constant: 5 kcal/molÅ^−2^), allowing the solvent and ions to move freely. This was followed by a 10 000 step minimization of the system without positional restraints on the peptide atoms, to remove steric clashes.^44^ The system was gradually heated from 0 to 300 K with positional restraints on the peptide (force constant: 25 kcal/molÅ^−2^) over 40 ps. Once the system reached 300 K, the positional restraints were gradually removed over 50 ps, and the system was equilibrated for another 100 ps without any positional restraints at 300 K, at constant pressure. A time constant of 0.1 ps was used for heat bath coupling for the system. The particle mesh Ewald (PME) method with a 9 Å real space cutoff was used to treat long‐range electrostatic interactions.^45^ The SHAKE algorithm was used to constrain bonds involving hydrogens.^46^ The resulting structures were used as the starting structures for production MD simulations. Production MD simulations were carried out for 500 ns in triplicates using pmemd, the CUDA module of Amber18. The same protocol for system preparation, minimization, equilibration, and production procedures was used for the phosphorylated peptide. The parameters for phosphorylated serine were taken from Homeyer et al.^47^

### 2.5 MD simulations of CK1δ complexes

The xleap module of AmberTools19 was used to prepare the CK1δ complexes for explicit solvent MD simulations. A three‐step minimization was carried out for the protein–peptide complexes using the sander module of the Amber11 package with the ff14SB force field. First, a 10 000 step energy minimization of the system was carried out, with positional restraints on the kinase and peptide (force constant: 5 kcal/molÅ^−2^) allowing ATP, the Mg^2+^ ions, solvent, and counterions to move freely. The second step of minimization was carried out over an additional 10 000 steps, this time with restraints on the kinase only (force constant: 5 kcal/molÅ^−2^), allowing the peptide, ATP, solvent, and ions to move freely. This was followed by a third 10 000 step minimization of the system without positional restraints, to remove steric clashes. The system was gradually heated from 0 to 300 K with positional restraints on the whole system (force constant: 25 kcal/molÅ^−2^) over 40 ps. Once the system reached 300 K, the positional restraints were gradually removed over 50 ps, and the system was equilibrated for another 100 ps without any positional restraints at 300 K, at constant pressure. The PME method with a 9 Å real space cutoff was used to treat long‐range electrostatic interactions.^45^ A time constant of 0.1 ps was used for heat bath coupling for the system. The SHAKE algorithm was used to constrain bonds involving hydrogens.^46^ The resulting structures were used as the starting structures for production MD simulations. Production MD simulations for 1 μs were carried out in triplicates using pmemd, the CUDA module of Amber18.

### 2.6 Accelerated MD simulations

Accelerated MD (aMD) was used in order to enhance conformational sampling of the systems, by adding a robust bias potential to the potential energy of the system in order to lower the height of local energy barriers between minima to allow the simulation to progress at a faster pace.^30^ In this study, dual‐boost aMD simulations were performed with boost energies added in both the total potential energy and the dihedral energy. The structures from explicit solvent production MD simulations for free peptides (after 500 ns) and CK1δ complexes (after 100 ns; described in the previous section) were used as starting structures for aMD. All simulations were carried out in explicit solvent at 300 K using the pmemd module of Amber18. For free peptides, production simulations were carried out for 250 ns, and for protein–peptide complexes production simulations were carried out for 500 ns. The same protocol was followed for production simulations of the mutant CK1δ complexes.

The convergence of the systems was evaluated by dividing the aMD trajectories into five equal segments and calculating the root mean square deviation (RMSD) of each segment using the first frame as reference. For the free peptide simulations, the initial aMD trajectories were divided into 50 ns segments, and for the bound peptides the trajectories were divided into 100 ns segments. Since both systems are intrinsically disordered the RMSD distribution plots have multiple peaks that represent different conformations, but the number of peaks decreases as a function of time, suggesting convergence (Figure S1A–D).

### 2.7 Binding energy calculations, energy decomposition, and alanine scanning

Molecular mechanics generalized born surface area and molecular mechanics Poisson Boltzmann surface area (MMPBSA) methods were used to calculate the binding free energies of the peptides to the kinase.^49^, ^50^ Binding free energies were calculated for each protein–peptide complex simulation. The effective binding energies were decomposed into contributions of individual residues using the MMPBSA energy decomposition method. The last 250 ns of the trajectories were used, sampling every 100 frames. Mbondi2 radii were used for these calculations.

Alanine scanning calculations were carried out to determine the residues within the kinase that contribute the most to binding to the peptides. Residues of the kinase that were located within 6 Å of the peptide were mutated to alanine with the exception of glycine. The polar contribution to the solvation free energy was calculated by using the generalized born method (igb = 5).

### 2.8 Two‐dimensional free energy surfaces

Two‐dimensional free energy surfaces (2DFESs) were plotted for the characterization of peptide energy landscapes from our simulations. The reaction coordinates selected were (i) radius of gyration (*R*_*g*_) of free peptide/peptide in the complex and (ii) end‐to‐end distance of free peptide/peptide in the complex.

### 2.9 Analysis

The cpptraj module of Amber18^51^ was used to calculate RMSD, *R*_*g*_, root mean square fluctuation (RMSF), solute–solute and solute–solvent hydrogen bond interactions, secondary structure using the DSSP method,^52^ end‐to‐end distance (measured between center of mass of the capping residues), distance between the γ‐phosphate of ATP and the side chain hydroxyl group of Thr18, and k‐means clustering. For the free peptide simulations, the first 100 ns were excluded from analysis for both normal and accelerated MD. For the aMD complex simulations, only the last 250 ns were considered. Visual molecular dynamics (VMD)^53^ and PyMOL^35^ were used for visualization of the simulations.

## 3 RESULTS

### 3.1 Conformational dynamics of free p53 peptides in solution

p53 TAD1 is highly dynamic and is known to adopt a helical conformation when bound to the hydrophobic pocket of MDM2 (PDB id 1YCR).^54^ In this work, the conformational dynamics of free p53 peptides in solution were explored by using the enhanced sampling method, aMD. Simulations of free peptides with and without phosphorylation were stable in solution. Due to the flexible nature of the free p53 peptides absolute convergence is not expected. However, examination of the RMSD distributions over 50 ns windows shows that the number of peaks (representing different conformational clusters) decrease over time for both unphosphorylated and phosphorylated peptides (Figure S1A,C), suggesting convergence. RMSD as a function of time (Figure S1E,F) again demonstrates convergence although the free peptides appear to occupy alternating minima in a pseudo‐periodic manner (Figure S1E).

**FIGURE 1:**
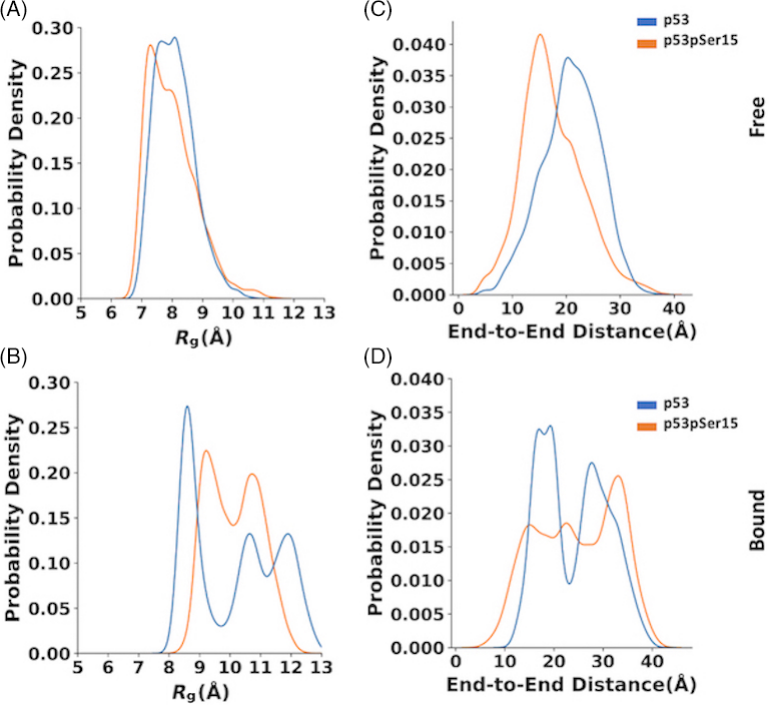
Distributions of radius of gyration (*R*_*g*_) and end‐to‐end distances for p53 and p53pSer15 peptides in their free and bound forms using Kernel Density Estimation (KDE). (A,C) Free peptide distributions. (B,D) Bound peptide (to CK1δ) distributions. p53 peptide distributions are colored in blue and p53pSer15 in orange.

**FIGURE 2:**
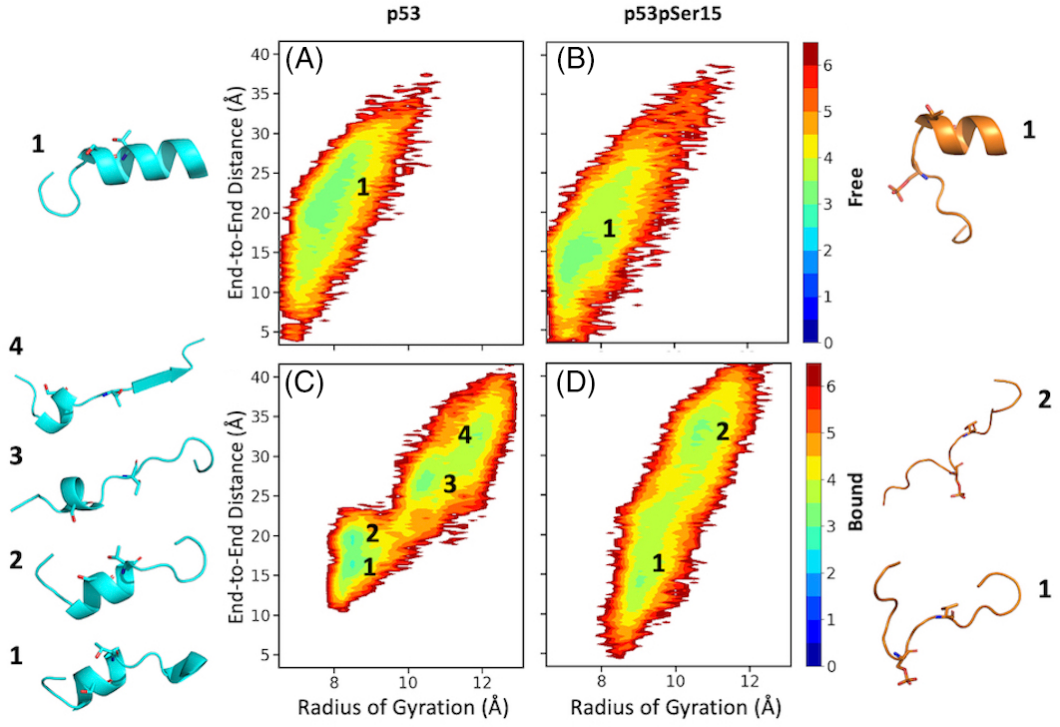
Two‐dimensional free energy landscape of free and bound p53 and p53pSer15 peptides as a function of radius of gyration (*R*_*g*_) and end‐to‐end distance. Panel (A) corresponds to conformations of the free p53 peptide. Panel (B) corresponds to conformations sampled in the free p53pSer15 peptide simulations. Panels (C,D) correspond to p53 and p53pSer15 peptide conformations bound to casein kinase 1δ, respectively. Representative conformations from each energy minimum are shown on the left and right sides of the plots and are numbered accordingly, with Ser15 and Thr18 residues shown as sticks.

The p53 and p53pSer15 peptides have similar R_g_ values ranging from 6 to 11 Å and from 6 to 12 Å, respectively. However, p53 has a broader peak at 8 Å whereas p53pSer15 has a narrower peak around 7.5 Å indicating a preference for slightly more expanded conformations in the unphosphorylated state (Figure 1A). The end‐to‐end distance distribution of p53 has a peak value around 20 Å while p53pSer15 peaks at 15 Å, values that are in line with observations from *R*_*g*_ (Figure 1C). *R*_*g*_ and end‐to‐end distance calculations suggest that p53 samples more extended conformations relative to p53pSer15. The 2DFESs plots for free peptides in solution were created with *R*_*g*_ on the x‐axis and end‐to‐end distance on the y‐axis. The p53 peptide has a large local energy minimum with an *R*_*g*_ value that spans from 8 to 9 Å and end‐to‐end distances range from 17 to 28 Å. The p53pSer15 peptide has an energy minimum with an *R*_*g*_ at 7.5 Å and an end‐to‐end distance between 10 and 17 Å (Figure 2A,B). The 2D energy surface analysis once more shows that the free p53 peptide is more extended in solution compared with p53pSer15.

### 3.2 Free p53 peptide retains helicity after Ser15 phosphorylation

**FIGURE 3:**
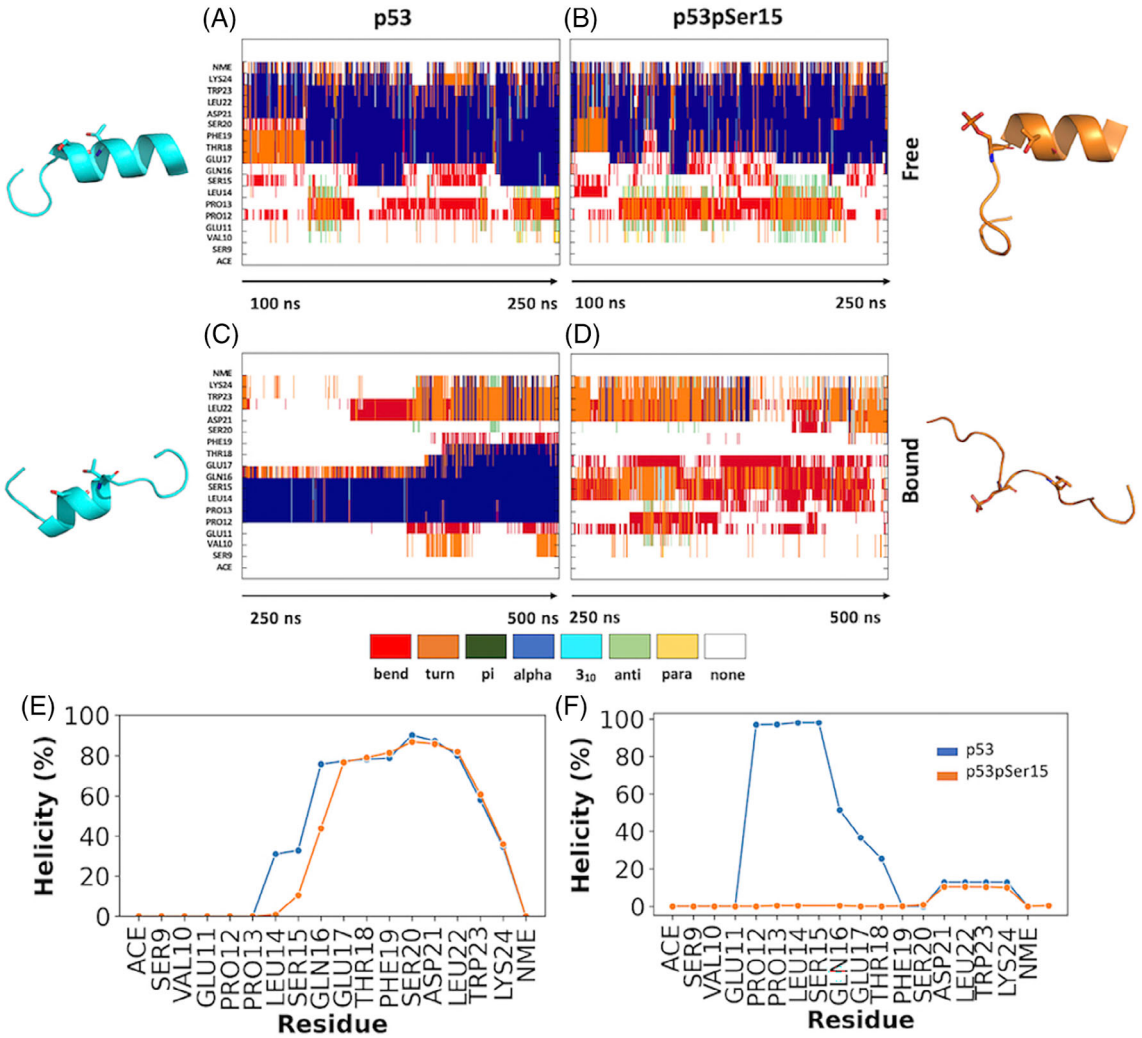
Secondary structure determination for p53 and p53pSer15 peptides in the free and bound states from accelerated molecular dynamics simulations. (A,B) Secondary structure per residue as a function of time (0.01 ns/frame) for free p53 and p53pSer15 peptides. (C,D) Secondary structure per residue as a function of time for p53 and p53pSer15 peptides bound to casein kinase 1δ. (E,F) Average percent helicity per residue in free and bound p53 and p53pSer15 peptides. p53 peptide is represented in blue and p53pSer15 peptide in orange.

The p53 peptide samples more helical conformations (41%) compared with p53pSer15 (37%). Helical conformations (α and 3_10_) are observed from residues Leu14 to Lys24 of p53 and Gln16 to Lys24 in p53pSer15 (Figure 3A,B,E). Ser15 is 33% helical in p53 and drops to 11% in p53pSer15. Phosphorylation at Ser15 affects helicity around the site as the negatively charged phosphate group prefers to be solvated, but the peptide maintains its helicity along the rest of the chain. Interactions from *i* to *i* + 4 were observed between backbone residues of Ser15‐Phe19 (48%), side chain and backbone residues of Leu14‐Thr18 (25% and 18%), and backbone residues of Thr18‐Leu22 (23%) in p53. The presence of the negatively charged phosphate results in attenuation of the hydrogen bonding interactions between the backbone of Ser15‐Phe19 (27%) and side chain of Leu14‐Thr18 (8%), whereas the Thr18‐Leu22 hydrogen bond interaction is not affected (22%).

### 3.3 Conformational dynamics of p53 peptides bound to CK1δ in solution

We next investigated the conformations sampled by the peptides when bound to CK1δ. Initial benchmark simulations of the modified crystal structure (data not shown) were carried out to test the stability of the complex with the selected force field. The complexes were stable using the ff14SB force field.^42^ The conformational dynamics of the p53 peptides with and without phosphorylation bound to CK1δ were studied using aMD. The convergence of the systems was evaluated by looking at their RMSD distributions as well as their RMSD with respect to time. The number of peaks in the RMSD distribution plot decrease over the simulation time suggesting convergence (Figure S1B,D). RMSD with respect to time indicates that while the systems are dynamic, they are mostly stable after the first 250 ns and undergo smaller motions compared with the free peptides (as expected; Figure S1F).

The range of *R*_*g*_ values for the bound p53 peptide from aMD simulations was between 7.5 and 13 Å with a major peak around 8.5 Å. The bound p53pSer15 peptide ranged from 8 to 13 Å with peaks around 9 and 11 Å (Figure 1B). The large *R*_*g*_ values signify that the phosphorylated peptide is more expanded than the unphosphorylated in the bound state. The same pattern is observed from end‐to‐end distance calculations for both the unphosphorylated and phosphorylated peptides. p53 has two broad peaks around 15 and 30 Å and p53pSer15 has a peak at 35 Å (Figure 1D). Free energy surface plots show that the p53 peptide has four local energy minima: one with an *R*_*g*_ value around 8.5 Å and an end‐to‐end distance of 15 Å, a second minimum with *R*_*g*_ value around 8.5 Å and an end‐to‐end distance of 20 Å, a third energy minimum with an *R*_*g*_ value at 10.5 Å and an end‐to‐end distance around 26 Å, and a fourth smaller minimum with an *R*_*g*_ at 12 Å and an end‐to‐end distance between 30 and 35 Å (Figure 2C). The free energy surface plot of p53pSer15 shows that there is a small local minimum with an *R*_*g*_ value at 9 Å and an end‐to‐end‐distance value around 14 Å and a second larger energy minimum with an *R*_*g*_ at 11 Å and an end‐to‐end distance between 30 and 35 Å (Figure 2D). The shift in local energy minima shows that phosphorylation causes the peptide to sample more extended conformations compared with the unphosphorylated peptide.

### 3.4 CK1δ bound p53 peptide loses helicity when Ser15 is phosphorylated

**FIGURE 4:**
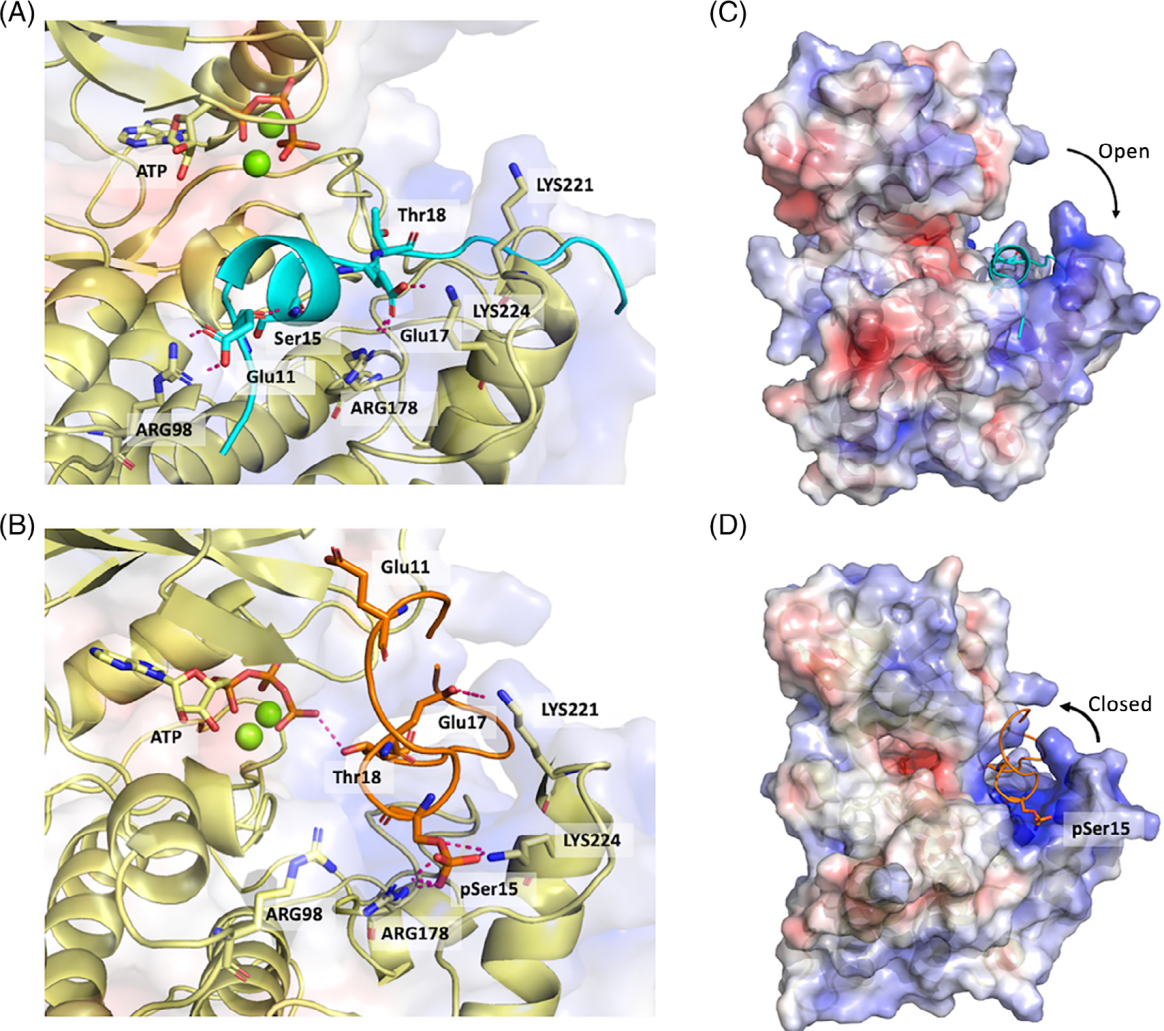
Structural basis for the binding of p53 peptides with and without phosphorylation on casein kinase 1δ (CK1δ). (A,B) CK1δ (yellow cartoon, transparent electrostatic surface) in complex with p53 (cyan) and p53pSer15 (orange). Protein–peptide H‐bond interactions within the binding pocket are represented by dashed lines. Mg^2+^ ions are shown as green spheres and adenosine triphosphate is shown in sticks. (C,D) Electrostatic potential surface of CK1δ (blue/red for positive/negative charge) in complex with p53 peptide is shown in cyan and p53pSer15 peptide is shown in orange.

The p53 favors more collapsed conformations than bound p53pSer15 when complexed to CK1δ. Secondary structure analysis of the bound peptides from aMD simulations shows that in addition to being more collapsed, the p53 peptide is more helical compared with p53pSer15. Phosphorylation at Ser15 destabilizes the helix observed in p53 and leads to a less structured peptide (Figure 3C,D,F). p53 has a total helicity (α, 3_10_) of 31%, whereas p53pSer15 is helical for only 4% of the simulation. The helix observed in the unphosphorylated peptide spans residues Pro12‐Thr18. An *i*, *i* + 4 interaction known to stabilize helices is observed between the backbone of residues Glu11 and Ser15 of p53 for 53% of the simulation (Figure 4A). Additional *i*, *i* + 4 interactions observed include the backbone of Pro12 and Gln16 (44%), and the backbone of Leu14 with the sidechain of Thr18 (32%).

In addition to intramolecular interactions, we also examined intermolecular interactions between the peptides and CK1δ to look for interactions that stabilize peptide binding. The sidechain of Ser15 in p53 interacts with Glu90 and Tyr179, whereas in p53pSer15, it almost exclusively interacts and forms a salt bridge with Arg178 which is located in the positively charged pocket of the kinase (Figures 4B and S2). Arg178 interacts with Glu17 in p53, while Glu17 also interacts with Lys224, occasionally forming salt bridges (Figure 4A). p53pSer15 samples a high population of disordered conformations and the phosphorylated, and negatively charged residues of the peptide are stabilized mainly by interactions with Arg178, Lys 221, and Lys 224 within the positively charged pocket of CK1δ (Figure 4B). Arg222 of CK1δ forms H‐bonds and in some cases salt bridges with Asp21 of p53 in both unphosphorylated and phosphorylated systems (Figure S3A,B). Arg98 is another positively charged residue near the catalytic pocket whose side chain only occasionally interacts with Glu11 in the p53pSer15 peptide due to the presence of several basic residues, but consistently forms a salt bridge with the side chain of Glu11 of p53 (Figure 4A). The sidechain of Lys130 which is also located near the catalytic pocket forms H‐bonds with the backbone of Gln16 of p53pSer15, but this interaction is not observed in the p53 peptide (Figure S4). At the catalytic site, CK1δ has a nonpolar patch compatible with a hydrophobic amino acid.^34^ This accommodates Phe19 of p53pSer15, being adjacent to Thr18, which in turn interacts with Gly175 of the kinase in both complexes (Figure S5A,B).

### 3.5 Effect of Ser15 phosphorylation on the structure of CK1δ

The introduction of a negatively charged phosphate group at Ser15 in the positively charged pocket of CK1δ also induces structural changes in the kinase. More specifically, the positively charged pocket of CK1δ closes due to the electrostatic attraction between the positively charged amino acids of the binding pocket and the negatively charged amino acids of the peptide (Figure 4D). This process is instigated by the presence of the negatively charged phosphate. Its absence in p53 results in the pocket remaining open (Figure 4C). Additionally, the p‐loop in the phosphorylated complex undergoes a conformational change that is accompanied by interactions between Ser19 and the backbone of Thr18 which also contributes to stabilizing the residue in a state that is appropriate for receiving the incoming phosphate from ATP (Figure S6). On the contrary, in the p53 complex, the side chain of Thr18 is not in the correct orientation for the phospho‐transfer reaction to take place and is further away compared with its location in p53pSer15.

### 3.6 Phosphorylation at Ser15 brings p53 Thr18 closer to ATP

**FIGURE 5:**
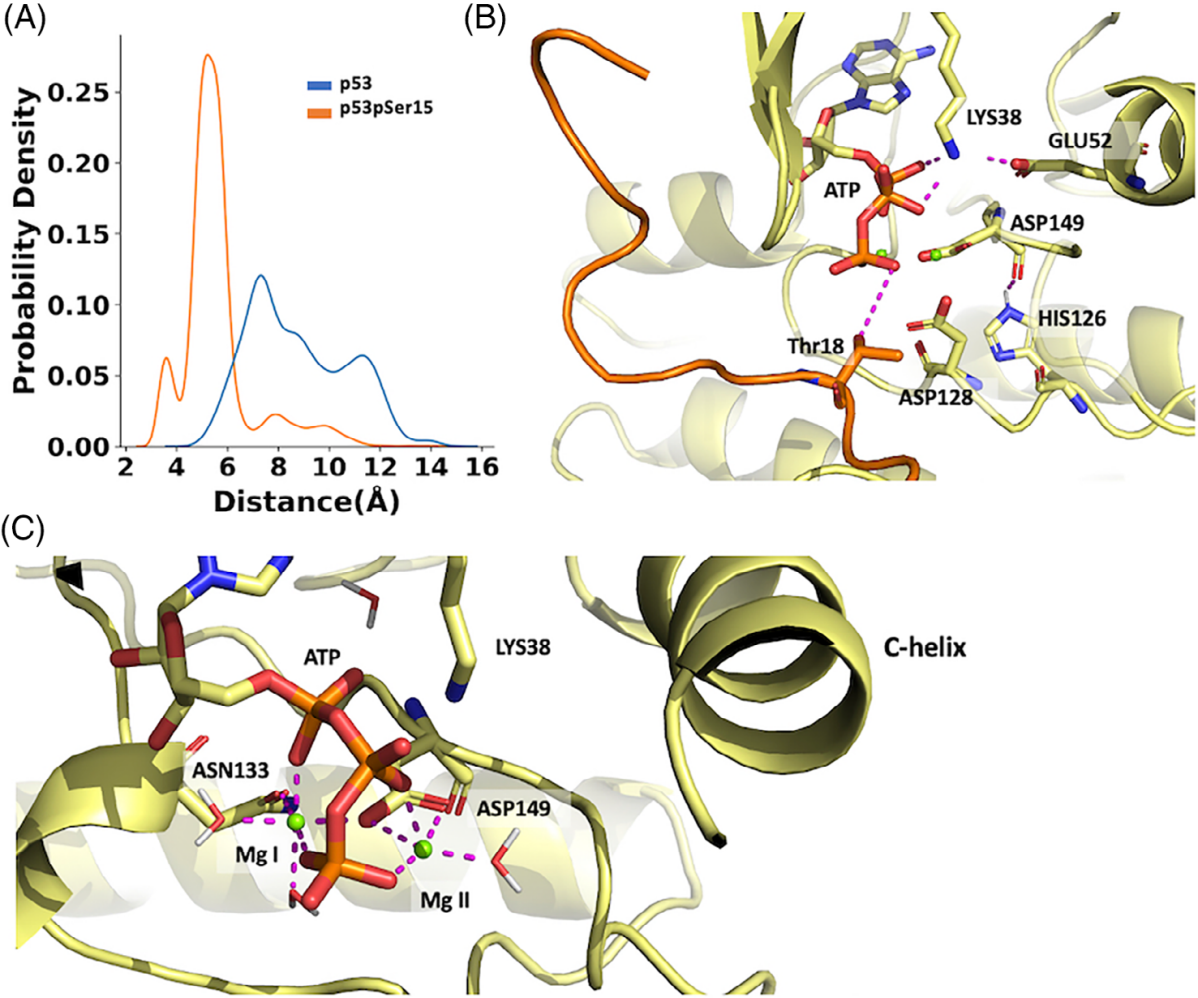
Thr18 is closer to the γ‐phosphate of adenosine triphosphate (ATP) when Ser15 is phosphorylated. (A) Probability density distribution of the distance from the side chain –OH of Thr18 to the γ‐phosphate of ATP. p53 and p53pSer15 peptides are colored in blue and orange, respectively. (B) Detailed interactions within the ATP binding pocket in the active kinase state. (C) Coordination of the 2 Mg^2+^ ions in the catalytic pocket of casein kinase 1δ (CK1δ). Mg I is coordinated by Asn133, Asp149, two oxygens from the α‐ and γ‐phosphates of ATP, and two water molecules. Mg II is coordinated with 2 H‐bonds from Asp149, two oxygens from the β‐ and γ‐phosphates of ATP and a water molecule. For (B,C), H‐bond interactions are shown as dashed lines. CK1δ is shown in yellow, the p53pSer15 peptide is shown in orange and the Mg^2+^ ions are shown as green spheres.

Next, we calculated the distance between the γ‐phosphate of ATP and the side‐chain oxygen of Thr18 for both p53 and p53pSer15 complexes. The distance for p53 spans a broad range between 4 and 16 Å, whereas for p53pSer15 the range is between 2 and 14 Å with a major peak at 5 Å (Figure 5A). The negatively charged phosphate in p53pSer15 and the accompanying disorder in the peptide clearly enable Thr18 to be placed close to and in the correct orientation relative to ATP for phosphate transfer.

Possible mechanisms of phosphate transfer include the base‐assisted mechanism (dissociative mechanism), and the substrate‐assisted mechanism (associative mechanism).^55^ During the base‐assisted mechanism of phosphate transfer, nucleophilic Thr18 would get deprotonated by Asp128 and become even more nucleophilic. In the substrate‐based mechanism, the hydroxyl proton of Thr18 would be transferred to a γ‐PO_4_ oxygen. Asp128 belongs to the conserved HRD (His‐Arg‐Asp) kinase motif, known to interact with the substrate in the active kinase state.^56^ As part of the phosphate transfer mechanism, Lys38 interacts with α‐ and β‐phosphate oxygens of ATP, while also interacting with Glu52 (Figure 5B). Asp149 is part of the conserved DFG (Asp‐Phe‐Gly) motif and it binds to the Mg^2+^ ion that directly interacts with an oxygen atom of the β‐phosphate of ATP. In the active kinase state Asp149 also interacts with the His from the HRD motif, in this case His126 (Figure 5B). The first Mg^2+^ ion interacts with two nonbinding oxygen atoms from the α‐ and γ‐phosphates of ATP, Asp149, Asn133, and two water molecules. The second Mg^2+^ ion is coordinated by a β‐ and a γ‐phosphate oxygen of ATP, a bidentate coordination with Asp149, and a water molecule (Figure 5C). A study by Recabarren et al.^55^ on CDK2, another Ser/Thr kinase describes the Mg^2+^ coordination states and phosphate transfer mechanism in more detail. Although our models could not unambiguously differentiate between the phosphate transfer mechanisms at play in the simulations, recent studies suggest that the base‐assisted mechanism is usually favored.^55^, ^57^, ^58^

### 3.7 Phosphorylated p53 peptide binds more strongly to CK1δ

Binding energy calculations on the MD trajectories were carried out using the MMPBSA method to characterize the energetics associated with the binding of p53 and p53pSer15 peptides to CK1δ. The binding of p53pSer15 (Δ*G* = −96.6 kcal/mol) to CK1δ was stronger than p53 (Δ*G* = −79.7 kcal/mol; Table 1). This is driven by greater van der Waals contributions that arise from the more extensive interactions between the extended conformation of p53pSer15 compared with the ordered conformation of p53, and by the electrostatic attraction between the positively charged pocket of CK1δ and the negatively charged phosphate of p53pSer15.

**TABLE 1:** Contributions made by van der Waals, electrostatic, and solvation energy components to the total estimated MMPBSA binding energy between p53/p53pSer15 peptides and CK1δ from aMD simulations

	vdW^a^ (kcal/mol)	EEL^b^ (kcal/mol)	EPB^c^ (kcal/mol)	Enpolar^d^ (kcal/mol)	Δ*G*_sol_ ^e^ (kcal/mol)	Δ*G*_total_ ^f^ (kcal/mol)
p53	−83.8	−839.3	858.6	−15.1	843.4	−79.7 (±1.2)
p53pSer15	−89.0	−1409.9	1417.7	−15.4	1402.2	−96.6 (±1.1)

Abbreviations: CK1δ, casein kinase 1δ; MD, molecular dynamics; MMPBSA, molecular mechanics Poisson Boltzmann surface area.

^a^ van der Waals contribution.

^b^ Electrostatic energy.

^c^ Polar contribution to the solvation free energy.

^d^ Nonpolar contribution to the solvation free energy.

^e^ Solvation free energy = EPB + Enpolar.

^f^ Final estimated binding free energy with standard error of mean.

**FIGURE 6:**
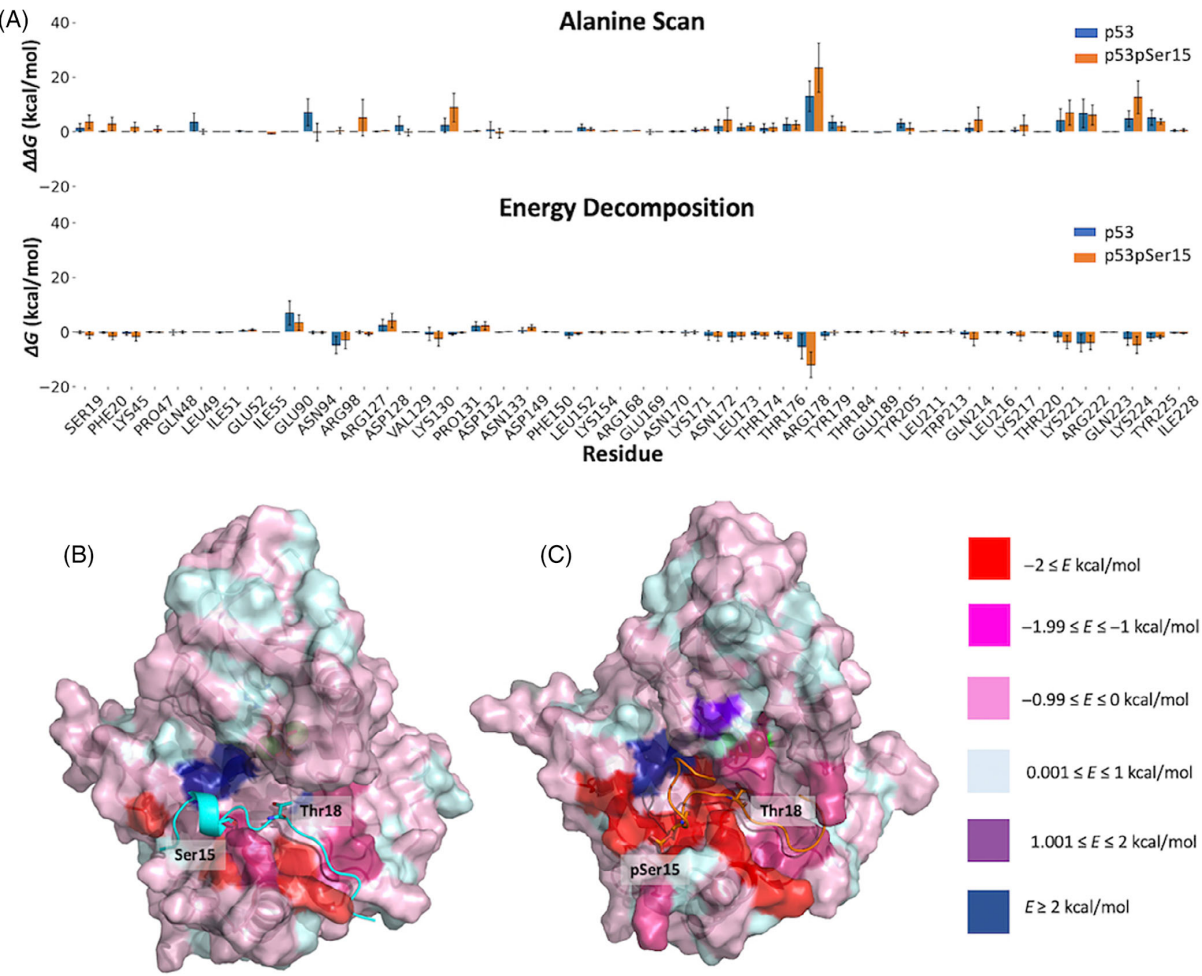
Individual energy contributions of casein kinase 1δ (CK1δ) residues to overall binding. (A) Per residue energy decomposition and alanine scan of CK1δ residues within 6 Å from peptides. CK1δ in complex with p53 is colored in blue and CK1δ in complex with p53pSer15 is colored in orange. (B,C) Surface of CK1δ in complex with p53 (cyan) and p53pSer15 (orange). The surface of CK1δ is color‐coded based on the residue energy contributions from energy decomposition calculations.

Decomposing the total energetics into contributions by residues revealed that Arg98, Arg178, Lys221, Arg222, and Lys224 make significant contributions to binding in both the p53 and p53pSer15 CK1δ complexes (Figure 6A–C). However, the contributions of Arg178, Lys221, and Lys224 are higher in the complex with p53pSer15 due to the presence of negatively charged phosphoserine. Other residues that contribute to binding are Lys130, Thr176, and Gln214 in the p53pSer15 complex and Tyr225 mainly in the p53 complex.

The individual contributions of different amino acids were also measured by performing alanine scanning. Alanine scanning was carried out for the p53 and p53pSer15 CK1δ complexes for all kinase residues within 6 Å of the peptides. All residues were mutated to Ala with the exception of Gly. The energy contribution, ΔΔ*G*, of the residues agreed with energy decomposition per residue calculations. In both methods, the positively charged residues in the catalytic pocket that contributed significantly to binding were Arg98, Lys130 (p53pSer15), Arg178, Lys221, Arg222, and Lys224 (Figure 6A).

### 3.8 Fuzzy binding of p53pSer15 peptide to CK1δ

**FIGURE 7:**
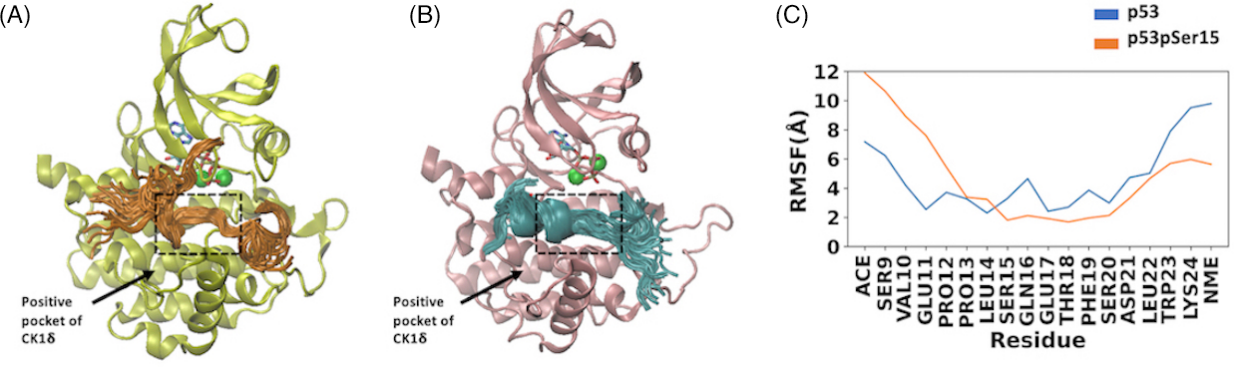
Fuzzy complexes of p53 and p53pSer15 peptides with casein kinase 1δ (CK1δ). (A) Representative conformations of the p53pSer15 peptide (orange) bound to CK1δ (yellow), resembling a flanking fuzzy complex. (B) Representative conformations of the p53 peptide (cyan) bound to CK1δ (pink), resembling a flanking fuzzy complex. For panels (A,B) Mg^2+^ ions are shown as green spheres and adenosine triphosphate is shown in sticks. The dashed line boxes focus on the location of residues Ser15–Ser20. (C) Root mean square fluctuation (RMSF) per residue for p53 (blue) and p53pser15 (orange) peptides.

The binding of p53pSer15 to CK1δ resembles a fuzzy complex since it retains conformational variability even after binding. CK1δ uses the pSer/pThr‐X‐X‐Ser/Thr motif in the linker region of the p53pSer15 peptide to recognize and bind to it, while it retains disordered flanking segments (Figure 7A). The linker region of p53pSer15 loses its helicity upon phosphorylation but binds more strongly to CK1δ due to electrostatic interactions that take place between the phosphorylated peptide and the kinase. The p53 peptide also binds to CK1δ and forms a fuzzy complex but interacts less strongly compared with the p53pSer15 peptide and samples different conformations (Figure 7B). RMSF was used to measure the average fluctuation per residue over time for the p53pSer15 and p53 peptides (Figure 7C). Residues Ser15‐Ser20 had lower RMSF values in the phosphorylated peptide. This suggests that the p53pSer15 peptide interacts more strongly with CK1δ and is more tightly bound compared with the unphosphorylated p53 peptide. The differences in fluctuations can be seen in Figure 7A,B where several overlapping conformations of each peptide are shown.

### 3.9 Impact of CK1δ mutations on p53 and p53pSer15 peptide binding

CK1δ residues Arg98, Lys130, Arg178, Lys221, Arg222, and Lys224 were mutated based on the results of alanine scanning. Individual aMD simulations for each mutation were carried out to test whether each residue's mutation to Ala would affect the peptide binding to CK1δ. Mutation simulations significantly affected the binding of p53 to the kinase while they had minimal effect on the binding of p53pSer15. Residue‐level decomposition analysis was carried out for each mutation and showed that the basic residues of CK1δ responsible for binding to the p53pSer15 peptide individually contribute significantly less when mutated to Ala; upon mutation the attenuation in interactions is compensated by neighboring positively charged residues. For example, when Arg178 is mutated to Ala, Lys224 has a larger energy contribution compared with the WT (Figure S7A). This pattern is also observed in simulations of mutations of K224A, whose interactions are compensated by Arg178 and Lys217 (Figure S7B). However, this is not the case in the p53 complex, where there is not enough negative charge and when one residue from the positively charged pocket is mutated the electrostatic interactions of the N‐terminus with the slightly less positive pocket are not strong enough to hold the peptide in place; indeed, in none of the mutations in CK1δ does unphosphorylated p53 stay in the binding pocket.

## 4 DISCUSSION

In this work, we used accelerated MD in an effort to understand why the p53 activating phosphorylation of Thr18 in TAD by CK1δ requires Ser15 phosphorylation as a prerequisite. We first studied the conformational dynamics of p53 and p53pSer15 peptides in their free forms and then in complex with CK1δ. MD simulations revealed that phosphorylation at Ser15 perturbs the structure of p53 peptide significantly when bound to CK1δ, while the structure of p53pSer15 remains partially helical when free in solution. On the other hand, p53 is mostly helical in its free form (between residues Leu14 and Lys24) and remains partly helical in the bound state (Pro12‐Thr18). This is in agreement with solution NMR studies which reported that free p53 TAD1 is helical between residues Thr18 to Leu26.^58^, ^59^ p53pSer15 lacks helical structure when bound to CK1δ and is localized closer to ATP compared with p53. The disordered nature of p53pSer15 increases its flexibility which enables it to bind tighter to CK1δ. Systems that are electrostatically driven are likely dominated by induced fit mechanisms such as the positively charged pocket of CK1δ and negatively charged pSer15 of the p53 peptide. Energetic analysis identified CK1δ residues Arg98, Lys130, Arg178, Lys221, and Arg222, Lys224 important for binding to p53pSer15. Arg178 and Lys224 had been speculated earlier to be involved in enabling interactions with acidic substrates.^60^

The conservation of the positively charged residues of the CK1δ pocket was examined across the CK1 family (not including its closest relatives tau tubulin kinases 1 and 2 and the vaccinia‐related kinases 1–3).^21^ Arg98, Lys130, and Arg178 are fully conserved across the CK1 subfamily. Arg222 and Lys224 are partially conserved (Figure S8A). Lys221 is not conserved across the CK1 subfamily (Figure S8A). We also investigated the conservation of these residues across species available in UniProt.^61^ All six positively charged residues in human CK1δ are fully conserved in mouse, rat, cattle, orangutan, African clawed frog, and Western clawed frog (Figure S8B). This clearly suggests that the sequential phosphorylation process with Ser15 getting phosphorylated first is likely conserved in evolution.

The motif pSer/pThr‐X‐X‐(X)‐Ser/Thr is the canonical consensus sequence for CK1δ substrates.^22^, ^23^ It is clear that the negatively charged phosphate at the −3 position is sequestered by a cationic patch on the kinase. There are also substrates that carry this motif, but with a negative charge in the form of Asp/Glu amino acids at the −3 position as these too can be sequestered by the cationic patch, albeit less strongly, because of the trigonal planar disposition of the carboxyl group and the less negative charge compared with the tetrahedral disposition and charge of the phosphate group.^62^ Substrates of CK1δ include TAp63α, where CK1δ phosphorylates four residues in a sequential manner having a Ser residue at the −3 position, with each newly phosphorylated residue acting as the priming site for the following phosphorylation event.^32^ Additionally, CK1δ phosphorylates two Ser residues on FOXO1a, with a “priming” phosphate on Ser at the −3 position.^63^ CK1δ also phosphorylates multiple Ser residues in the acidic domain of MDM2 using acidic amino acids as priming residues.^64^ BID contains a cluster of acidic residues upstream of its Ser residue phosphorylated by CK1, and once phosphorylated can serve as the priming residue for additional phosphorylation events.^65^, ^66^ Furthermore, YAP a protein increasingly recognized for its role in health/disease that is part of the HIPPO pathway (YAP/TEAD interaction), gets phosphorylated by Lats on Ser381, which provides a priming signal for CK1δ to phosphorylate Ser384 and Ser387.^67^ It is interesting that the associated YAP sequence is ^379^DESTDSGLSM^388^ and phosphorylation of Ser381 would result in a long‐negative potential subtended by Asp, Glu, and pSer, that likely translates into higher affinity of the peptide. Detailed lists of CK1δ substrates can be found in comprehensive reviews on the CK1 family of kinases.^21^, ^66^ In addition, it also appears that a hydrophobic residue located at the +1 or +2 position may serve to further anchor the peptide by interacting with a hydrophobic patch on CK1δ that includes Gly175 (this residue and the region are evolutionarily conserved in the CK1 family; Figure S8). For example, in p53 the residue interacting with the hydrophobic patch of CK1δ is Phe19, in FOXO1 is Gly326 (phosphorylation sites are S322 and S325), and in YAP Ser384 and Ser387 are followed by hydrophobic residues Gly385 and Leu388, respectively. Indeed, in the case of p53, the contribution of Phe19 to the total interaction energy is approximately −4.9 kcal/mol in the phosphorylated state and approximately −3.5 kcal/mol in the unphosphorylated state. The higher stability in the phosphorylated state suggests that this hydrophobic interaction may be a second anchor point, together with the acidic region, to hold the substrate for phosphorylation.

IDPs such as p53 TAD1, are complex systems and often have multiple binding partners. The structure of IDPs fluctuates as they bind to different partners. TAD1 of p53 has a helical conformation when bound to MDM2, and it was found that free p53 TAD1 peptides retain their helicity in solution and bind to MDM2 through conformational selection.^69^ Our observations on the helicity of free p53 TAD1 peptides from residue Gln16 onwards are consistent with the study of Yadahalli et al.^69^ Our molecular modeling studies showed that p53 TAD1 also partially retains its helicity when bound to CK1δ. However, p53 TAD1 loses helicity upon Ser15 phosphorylation, and its binding mechanism appears to be induced fit.

In summary, phosphorylation of Thr18 in p53 TAD1 by CK1δ requires Ser15 to be phosphorylated first to enable sequestration of the p53pSer15 peptide to the positively charged pocket of CK1δ. This interaction is mediated by a network of conserved positive charges in CK1δ and the negatively charged p53pSer15 peptide. Phosphorylated Ser15 serves as the initial anchor point, which enables the remaining peptide to morph itself onto the surface of CK1δ, forming a fuzzy complex. This places the Thr18 hydroxyl group within close proximity and in the right orientation relative to the γ‐phosphate of ATP for efficient transfer of the phosphate to Thr18. Our models suggest that a hydrophobic interaction at the +1 position of Thr18 may further contribute to anchoring the peptide. The combination of these results along with additional experimental studies focusing on p53 TAD1 and its binding to various partners could further decipher the activation mechanism of p53 by multisite phosphorylation which could be exploited for drug discovery.

## FUNDING INFORMATION

This work was supported by a joint PhD studentship from the University of Manchester and A*STAR Singapore.

## CONFLICT OF INTERESTS

Chandra S. Verma and Srinivasaraghavan Kannan are founders of Sinopsee Therapeutics and Aplomex.

## Supporting information


**FIGURE S1.** Root mean square deviation (RMSD) of free p53/p53pSer15 peptides and p53/p53pSer15 peptides in complex with casein kinase 1δ (CK1δ). (A,C) Free p53 and p53pSer15 peptide RMSD distributions calculated using Kernel Density Estimation (KDE). Accelerated molecular dynamics (aMD) trajectories were divided into five 50 ns segments. Exactly 0–50 ns are colored in blue, 50–100 ns are in orange, 100–150 ns are in green, 150–200 ns in red, and 200–250 ns in purple. (B,D) Bound p53 and p53pSer15 peptide (to CK1δ) distributions calculated using KDE. Trajectories were divided into 5100 ns segments. Exactly 0–100 ns are colored in blue, 100–200 ns are in orange, 200–300 ns are in green, 300–400 ns in red, and 400–500 ns in purple. (E) RMSD as a function of time for free p53 and p53pSer15 peptides, in blue and orange, respectively. (F) RMSD as a function of time for complexes of p53 and p53pSer15 peptides bound to CK1δ in blue and orange, respectively. For (E,F), the number of frames is on the x‐axis and RMSD is on the y‐axis.
**FIGURE S2.** H‐bond interactions between Glu90, Tyr179, and Tyr205 of casein kinase 1δ (yellow) and Ser15 of p53 peptide (cyan), shown as dashed lines. Mg^2+^ ions are shown as green spheres. H‐bond interactions are shown as dashed lines.
**FIGURE S3.** H‐bond interactions between Arg222 of casein kinase 1δ (CK1δ) and Asp21 of p53 TAD1 peptide. (A) CK1δ is shown in yellow, p53 peptide is shown in cyan and H‐bond interactions are represented by dashed lines. (B) CK1δ is shown in yellow, p53pSer15 peptide is shown in orange and H‐Bond interactions are shown as dashed lines. For (A,B) Mg^2+^ ions are shown as green spheres.
**FIGURE S4.** H‐bond interaction between Lys130 of casein kinase 1δ (yellow) and the backbone carbonyl Gln16 of p53pSer15 peptide, represented by dashed lines. The Mg^2+^ ions are shown as green spheres.
**FIGURE S5.** H‐bond interaction at the +1 position of Thr18 between Phe19 and Gly175 of casein kinase 1δ (CK1δ). (A) CK1δ (yellow) in complex with p53 peptide (cyan). (B) CK1δ (yellow) in complex with p53pSer15 peptide (orange). Mg^2+^ ions are represented by green spheres. H‐bond interactions are shown as dashed lines.
**FIGURE S6**. H‐bond interaction between Ser19 of casein kinase 1δ (CK1δ) and Thr18 of p53pSer15 peptide. CK1δ is shown in yellow and p53pSer15 is shown in orange. Mg^2+^ ions are represented by green spheres. H‐bond interactions are shown as dashed lines.
**FIGURE S7.** Energy contribution per residue from WT and Ala mutation simulations of casein kinase 1δ (CK1δ)–p53pSer15 complex. Mutation of one positively charged residue of CK1δ leads to another positively charged residue taking over to hold p53pSer15 in place. (A) Per residue energy contribution of CK1δ–R178A. (B) Per residue energy contribution of CK1δ–K224A. For (A,B), mutated residues are circled in green. Energy contributions of WT CK1δ are colored in blue and energy contributions of mutated CK1δ complexes are colored in red.
**FIGURE S8.** Alignment of (A) CK1 subfamily and (B) casein kinase 1δ (CK1δ) across different species, using the program ClustalW 1,2. Conserved amino acids within the positive pocket of CK1δ are highlighted in blue, amino acids with highly similar properties are highlighted in green, and not conserved amino acids are highlighted in purple.

## Data Availability

All simulation data can be provided upon request.
