# Supplementary material for: Activation of p53: How phosphorylated Ser15 triggers sequential phosphorylation of p53 at Thr18 by CK1δ
Source: Proteins. 2022 Jul 14;90(12):2009–22. doi: 10.1002/prot.26393 (PMC9796392; doi:10.1002/prot.26393)
Supplement: Supplementary file 1 — FIGURE S1. Root mean square deviation (RMSD) of free p53/p53pSer15 peptides and p53/p53pSer15 peptides in complex with casein kinase 1δ (CK1δ). (A,C) Free p53 and p53pSer15 peptide RMSD distributions calculated using Kernel Density Estimation (KDE). Accelerated molecular dynamics (aMD) trajectories were divided into five 50 ns segments. Exactly 0–50 ns are colored in blue, 50–100 ns are in orange, 100–150 ns are in green, 150–200 ns in red, and 200–250 ns in purple. (B,D) Bound p53 and p53pSer15 peptide (to CK1δ) distributions calculated using KDE. Trajectories were divided into 5100 ns segments. Exactly 0–100 ns are colored in blue, 100–200 ns are in orange, 200–300 ns are in green, 300–400 ns in red, and 400–500 ns in purple. (E) RMSD as a function of time for free p53 and p53pSer15 peptides, in blue and orange, respectively. (F) RMSD as a function of time for complexes of p53 and p53pSer15 peptides bound to CK1δ in blue and orange, respectively. For (E,F), the number of frames is on the x‐axis and RMSD is on the y‐axis. FIGURE S2. H‐bond interactions between Glu90, Tyr179, and Tyr205 of casein kinase 1δ (yellow) and Ser15 of p53 peptide (cyan), shown as dashed lines. Mg2+ ions are shown as green spheres. H‐bond interactions are shown as dashed lines. FIGURE S3. H‐bond interactions between Arg222 of casein kinase 1δ (CK1δ) and Asp21 of p53 TAD1 peptide. (A) CK1δ is shown in yellow, p53 peptide is shown in cyan and H‐bond interactions are represented by dashed lines. (B) CK1δ is shown in yellow, p53pSer15 peptide is shown in orange and H‐Bond interactions are shown as dashed lines. For (A,B) Mg2+ ions are shown as green spheres. FIGURE S4. H‐bond interaction between Lys130 of casein kinase 1δ (yellow) and the backbone carbonyl Gln16 of p53pSer15 peptide, represented by dashed lines. The Mg2+ ions are shown as green spheres. FIGURE S5. H‐bond interaction at the +1 position of Thr18 between Phe19 and Gly175 of casein kinase 1δ (CK1δ). (A) CK1δ (yellow) in complex wit [file PROT-90-2009-s001.pdf]

Activation of p53: how phosphorylated Ser15 triggers sequential phosphorylation of p53 at Thr18 by CK1δ

Sonia T. Nicolaou[1,2], Srinivasaraghavan Kannan[2], Jim Warwicker[1], Chandra S. Verma[2,3,4,*]

[1]School of Biological Sciences, Faculty of Biology, Medicine and Health, Manchester Institute of Biotechnology, University of Manchester, Manchester M1 7DN, UK [2]Bioinformatics Institute, Agency for Science, Technology, and Research (A*STAR), Singapore 138671, Singapore [3]School of Biological Sciences, Nanyang Technological University, 60 Nanyang Drive, Singapore 637551 [4]Department of Biological Sciences, National University of Singapore, 14 Science Drive 4, Singapore 117543

* Correspondence: Chandra S. Verma, Phone: +65 64788293, Fax: +65 64789048, Email: chandra@bii.a-star.edu.sg

SUPPLEMENTARY MATERIAL

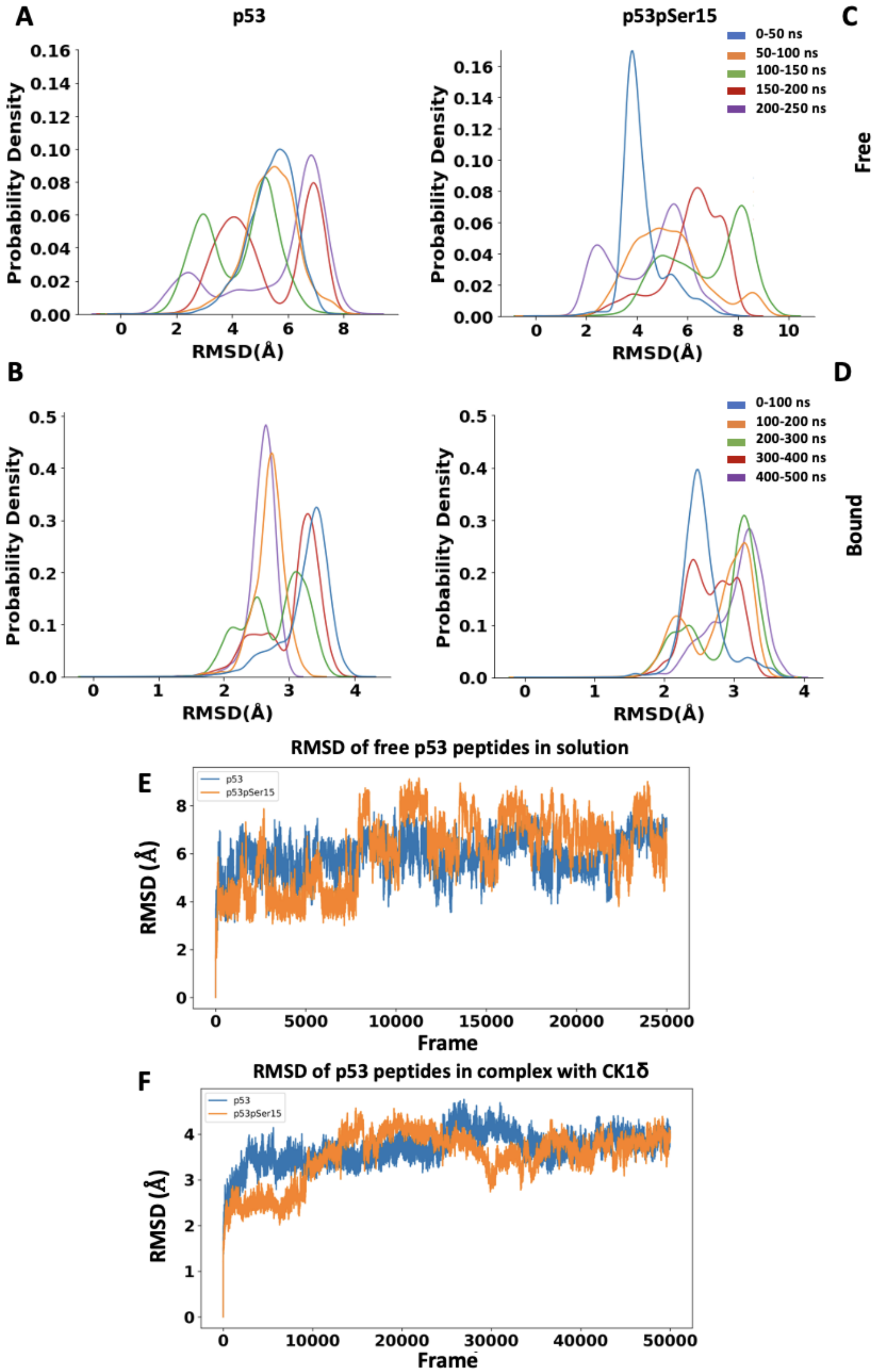

Supplementary Figure S1. Root mean square deviation (RMSD) of free p53/p53pSer15 peptides and p53/p53pSer15 peptides in complex with CK1δ. (**A** and **C**) Free p53 and p53pSer15 peptide RMSD distributions calculated using Kernel

Density Estimation (KDE). aMD trajectories were divided into 5 50 ns segments. 0-50 ns are colored in blue, 50-100 ns are in orange, 100-150 ns are in green, 150-200 ns in red, and 200-250 ns in purple. (**B** and **D**) Bound p53 and p53pSer15 peptide (to CK1δ) distributions calculated using KDE. Trajectories were divided into 5 100 ns segments. 0-100 ns are colored in blue, 100-200 ns are in orange, 200-300 ns are in green, 300-400 ns in red, and 400-500 ns in purple. (**E**) RMSD as a function of time for free p53 and p53pSer15 peptides, in blue and orange respectively. (**F**) RMSD as a function of time for complexes of p53 and p53pSer15 peptides bound to CK1δ in blue and orange, respectively. For panels **E** and **F,** the number of frames is on the x-axis and RMSD is on the y-axis.

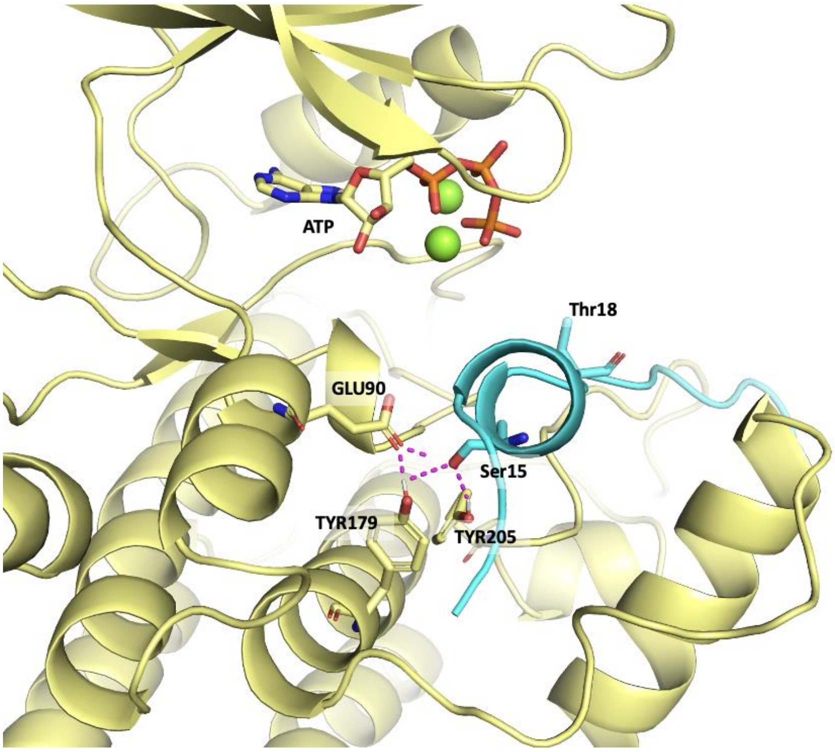

Supplementary Figure S2. H-bond interactions between Glu90, Tyr179 and Tyr205 of CK1δ (yellow) and Ser15 of p53 peptide (cyan), shown as dashed lines. Mg^{2+} ions are shown as green spheres. H-bond interactions are shown as dashed lines.

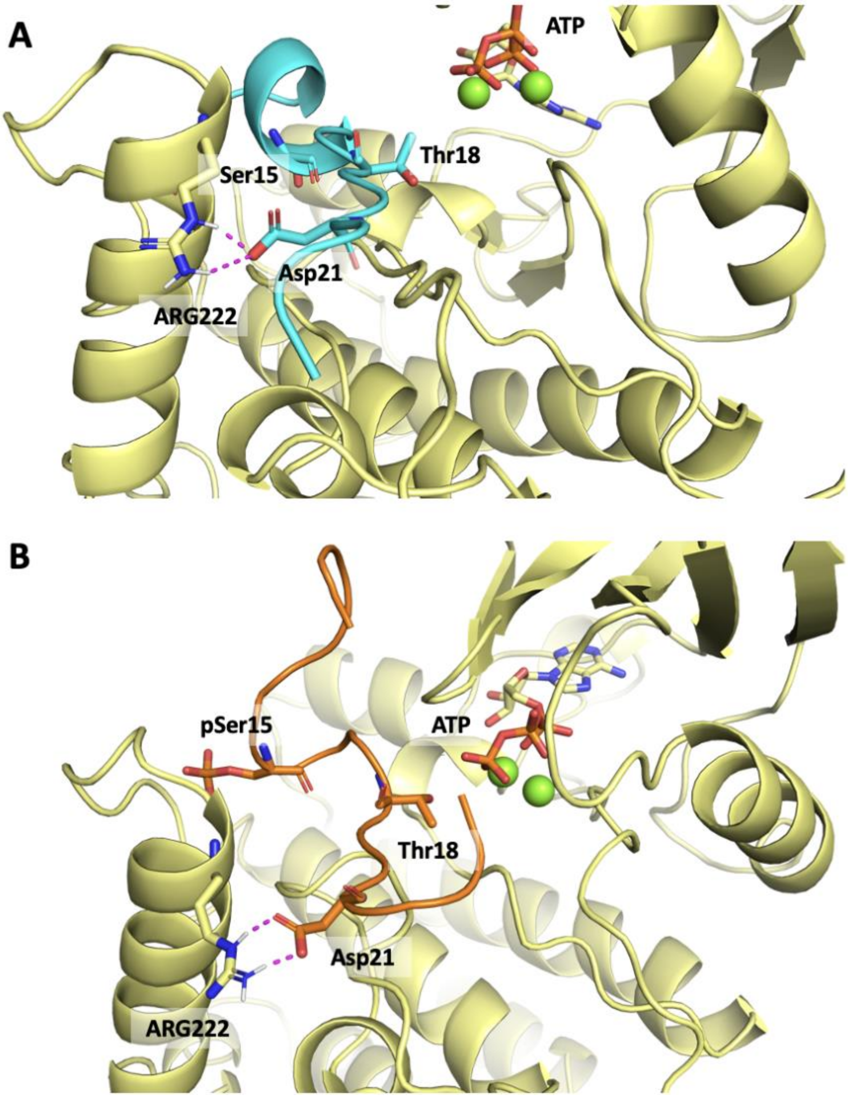

Supplementary Figure S3. H-bond interactions between Arg222 of CK1δ and Asp21 of p53 TAD1 peptide. (**A**) CK1δ is shown in yellow, p53 peptide is shown in cyan and H-bond interactions are represented by dashed lines. (**B**) CK1δ is shown in yellow, p53pSer15 peptide is shown in orange and H-Bond interactions are shown as dashed lines. For panels **A** and **B** Mg^{2+} ions are shown as green spheres.

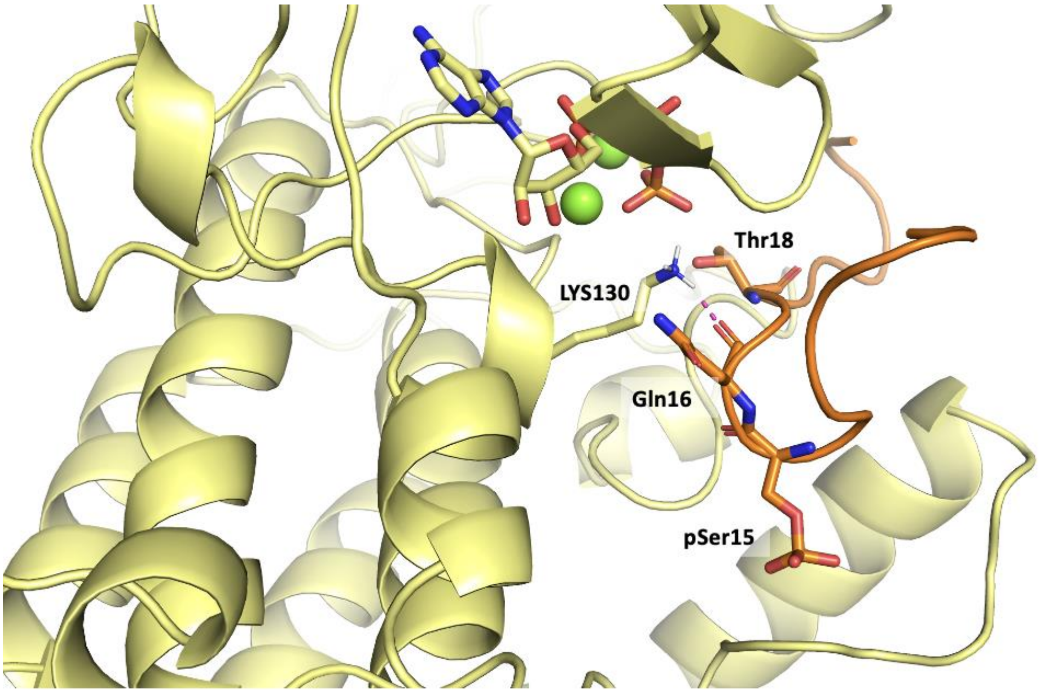

Supplementary Figure S4. H-bond interaction between Lys130 of CK1δ (yellow) and the backbone carbonyl Gln16 of p53pSer15 peptide, represented by dashed lines. The Mg^{2+} ions are shown as green spheres.

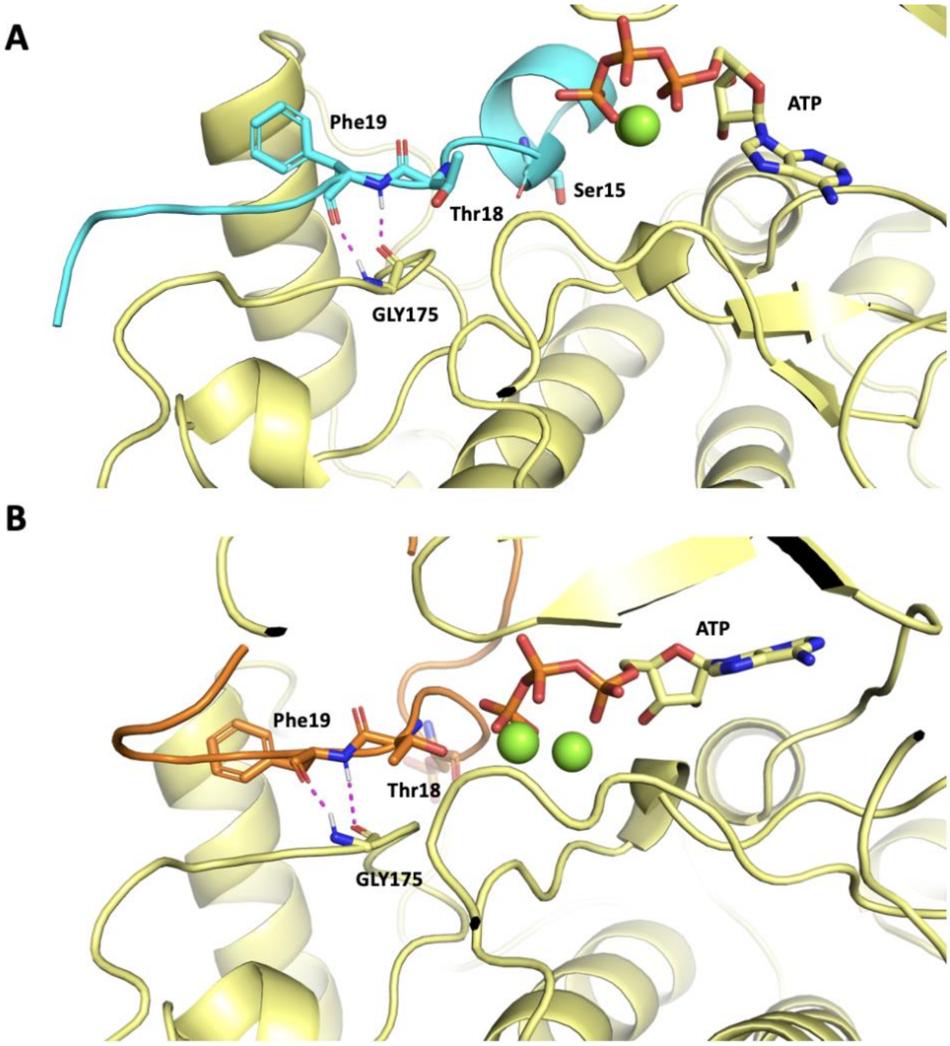

Supplementary Figure S5. H-bond interaction at the +1 position of Thr18 between Phe19 and Gly175 of CK1δ. (**A**) CK1δ (yellow) in complex with p53 peptide (cyan). (**B**) CK1δ (yellow) in complex with p53pSer15 peptide (orange). Mg^{2+} ions are represented by green spheres. H-bond interactions are shown as dashed lines.

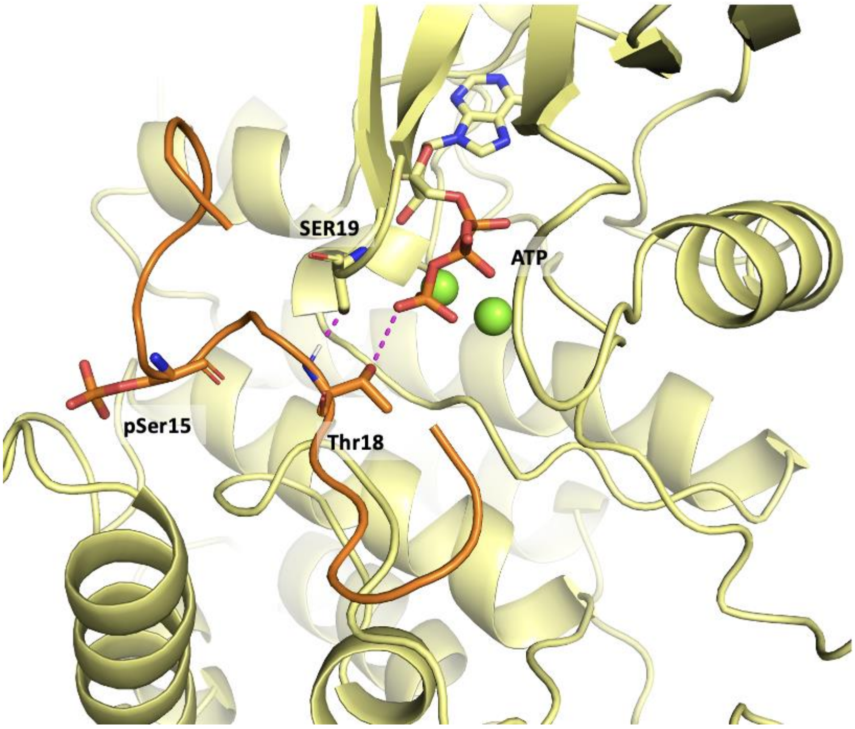

Supplementary Figure S6. H-bond interaction between Ser19 of CK1δ and Thr18 of p53pSer15 peptide. CK1δ is shown in yellow and p53pSer15 is shown in orange. Mg^{2+} ions are represented by green spheres. H-bond interactions are shown as dashed lines.

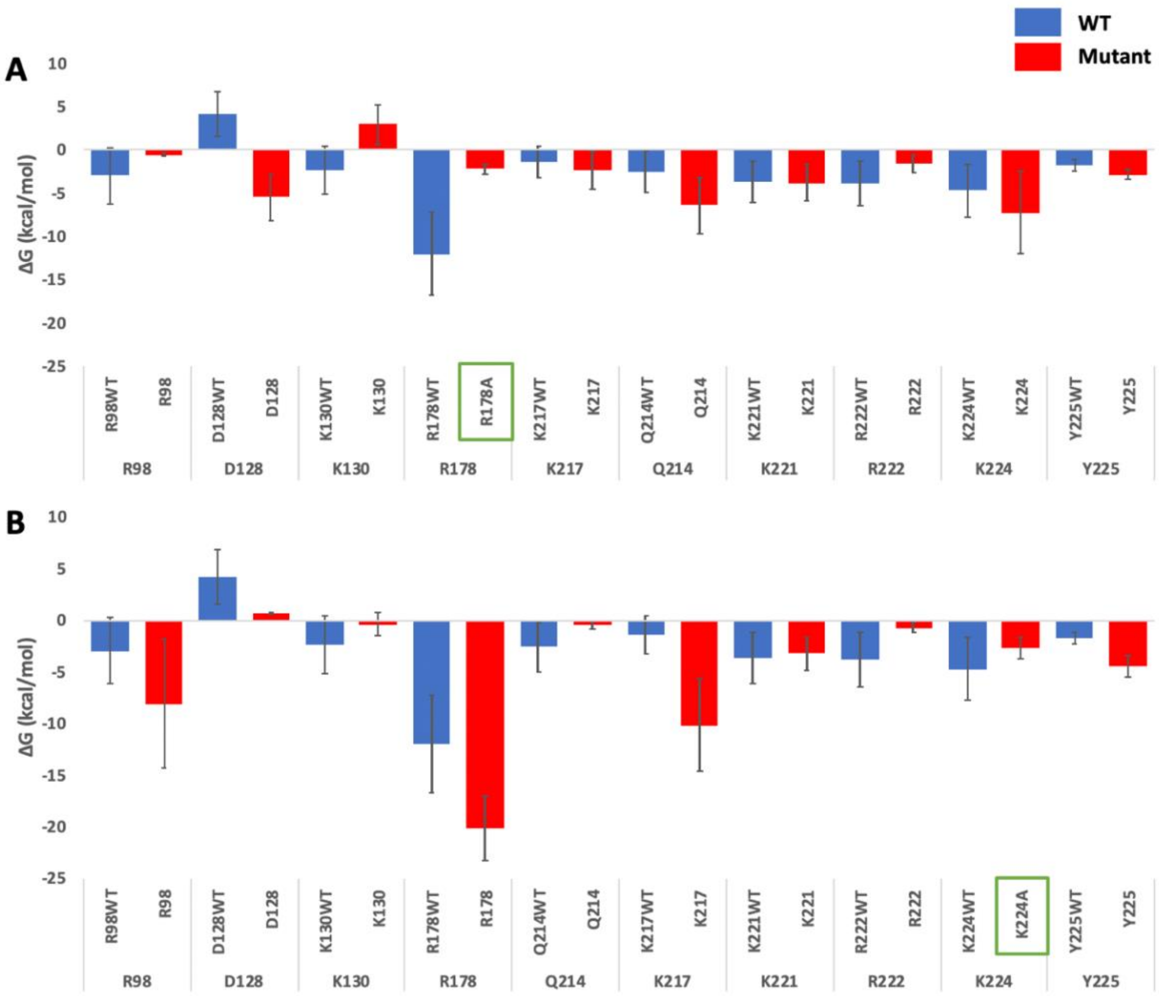

Supplementary Figure S7. Energy contribution per residue from WT and mutation simulations of CK1δ-p53pSer15 complex. Mutation of one positively charged residue of CK1δ leads to another one taking over to hold p53pSer15 in place. (**A**) Per residue energy contribution of CK1δ-R178A. (**B**) Per residue energy contribution of CK1δ-K224A. For panels **A** and **B**, mutated residues are circled in green. Energy contributions of WT CK1δ is colored in blue and energy contributions of mutated CK1δ complexes are colored in red.

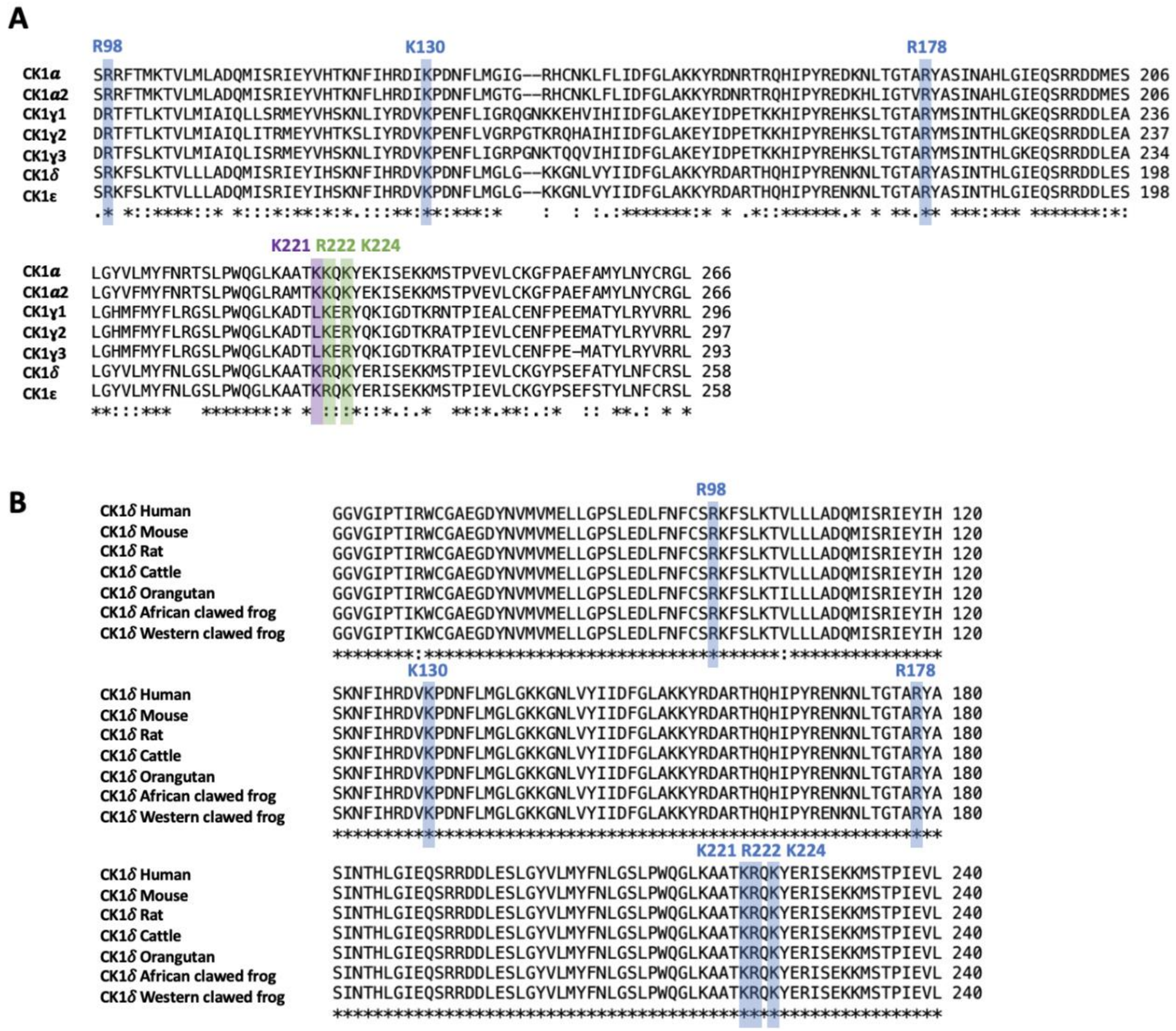

Supplementary Figure S8. Alignment of (**A**) CK1 subfamily and (**B**) CK1δ across different species, using the program ClustalW [1,2]. Conserved amino acids within the positive pocket of CK1δ are highlighted in blue, amino acids with highly similar properties are highlighted in green, and not conserved amino acids are highlighted in purple.

References

1. Larkin MA, Blackshields G, Brown NP, et al. Clustal W and Clustal X version 2.0. *Bioinformatics*. 2007;23(21):2947-2948.
2. Goujon M, McWilliam H, Li W, et al. A new bioinformatics analysis tools framework at EMBL-EBI. *Nucleic Acids Res*. 2010;38(Web Server issue).
